# Enhanced detection of equine strongyles: Insights from morphological and nemabiome metabarcoding approaches in northern Iran

**DOI:** 10.1111/evj.70120

**Published:** 2025-11-29

**Authors:** Sina Mohtasebi, Sangwook Ahn, Mahan Karimi, Mohammad Saberi, John S. Gilleard, Jocelyn Poissant

**Affiliations:** ^1^ Faculty of Veterinary Medicine University of Calgary Calgary Alberta Canada; ^2^ Department of Medical Parasitology and Mycology, School of Medicine Guilan University of Medical Sciences Rasht Iran; ^3^ Department of Medical Parasitology and Mycology, School of Public Health Tehran University of Medical Sciences Tehran Iran

**Keywords:** cyathostomin, DNA metabarcoding, horse, ITS2, mixed infection, molecular diagnosis, *Strongylus vulgaris*

## Abstract

**Background:**

Strongyles pose significant health concerns for equids globally. Strongyles, comprising over 60 species, can lead to severe morbidity and mortality, with *Strongylus vulgaris* posing higher risks due to its migratory behaviour. Routine diagnostic methods, such as faecal egg counts, lack species‐level resolution, while traditional morphological techniques require advanced expertise. DNA metabarcoding offers a high‐throughput alternative.

**Objectives:**

To characterise the diversity of strongyles infecting horses in northern Iran and evaluate how age, sex, diagnostic methods and host population influence community composition.

**Study Design:**

Cross‐sectional.

**Methods:**

Strongyle communities were studied across four locations. At two farms, subsets of horses were analysed either by morphological identification of adult worms or by ITS2 metabarcoding of larval cultures. Morphological identification was performed on 1476 adult worms recovered from 23 horses at two farms (Rezvanshahr and Gisum). In parallel, ITS2 nemabiome metabarcoding was applied to pools of ~2500 L3 larvae from faeces of 25 untreated horses. Community composition was analysed using dissimilarity indices (Jaccard, Bray–Curtis), PERMANOVA and generalised linear models to assess the effects of farm, method, age and sex.

**Results:**

Thirty‐three species were detected across both methods. DNA metabarcoding identified more species and 11 species were recorded in Iran for the first time. Strongyle community composition varied significantly among locations, including between resident and non‐resident horses at the riding club, and between diagnostic methods. Neither horse age nor sex explained variation. *S. vulgaris* was prevalent across the majority of locations, potentially due to inconsistent treatment.

**Main Limitations:**

Morphological and nemabiome identifications were conducted on different subsets of horses in the same location, precluding direct within‐individual comparisons. The study relied on owner‐reported information about horse characteristics and management practices.

**Conclusion:**

These findings provide new insights into strongyle diversity in northern Iran and highlight the value of molecular diagnostics for equine parasite surveillance and control.

## 1 INTRODUCTION

Strongyle nematodes are the predominant cause of parasitic infections in horses worldwide, and mixed infections involving multiple strongyle species are common in both domestic and feral populations.^1^, ^2^ These infections can lead to complex pathologies and require precise diagnostic methods for effective control.^3^ Traditional diagnostic methods, such as faecal egg counts (FEC), are widely used to test for the presence of parasitic infections in domestic and companion animals.^4^ However, these methods have limitations, including the inability to differentiate species having identical egg morphologies. With pathology^5^, ^6^, ^7^ and the emergence of anthelmintic resistance^8^ being species‐specific, species‐level diagnostic approaches are necessary to develop targeted parasite management practices.^9^

Molecular diagnostic methods have emerged as powerful tools for the accurate identification of parasitic species in mixed infections.^10^, ^11^ Techniques such as DNA metabarcoding allow for the comprehensive characterisation of parasite communities, offering insights into species composition and infection dynamics that are not possible with traditional methods.^12^ This is particularly important for managing parasitic infections in livestock, where the accurate identification of parasites can inform targeted interventions and improve animal health outcomes.^13^ The development of molecular assays using DNA metabarcoding, known as ‘nemabiome’ metabarcoding, has paved the way for non‐invasive identification of mixed parasitic infections using the internal transcribed spacer‐2 (ITS2) gene region.^12^ The application of nemabiome metabarcoding has supported studies on mixed infections in domestic and wild populations, such as sheep,^14^ cattle,^12^ horses,^15^ primates,^16^ reindeer^17^ and roe deer.^18^

Equids (such as horses, donkeys and zebras) are host to over 80 species of parasitic nematodes, many of which are significant health concerns.^19^ A large majority of equine nematode parasites belong to a single family, Strongylidae, with 64 species in 19 genera (commonly known as strongyles).^20^ These parasites are classified into two subfamilies, including 14 species belonging to the subfamily Strongylinae (large strongyles) and 50 species belonging to the subfamily Cyathostominae (small strongyles or cyathostomins).^20^ Strongyle infection can lead to severe health issues in horses at any age with high morbidity and mortality.^19^ Among large strongyles, four species, including *Strongylus vulgaris, Strongylus edentatus, Strongylus equinus* and *Strongylus asini* are known as migratory strongyles with the capability of causing more severe pathology.^19^ Migration of *S. vulgaris* larvae, considered the most pathogenic species,^21^ can cause arteritis of the cranial mesenteric artery, leading to thromboembolism and intestinal infarction, which can be fatal.^19^ Cyathostomins can also contribute to clinical complications, such as larval cyathostominosis,^22^ and are of particular concern as anthelmintic resistance has been detected in multiple species and may reduce the efficacy of commonly used treatments.^23^ The combination of pathogenic potential, species‐specific drug resistance and changing management practices makes understanding the population dynamics of these nematodes a priority for equine health worldwide.

Routine diagnosis of strongyle infections in horses typically relies on faecal egg counts (FECs).^4^, ^24^ A major limitation of FECs is that egg morphology does not allow different strongyle species to be differentiated.^20^ Identifying species is important because horses are typically infected by multiple strongyle species at once, and the prevalence and intensity of infections by various species differ among individuals.^25^ Although identification of *Strongylus* spp. in their infective larval stages is possible through microscopy following larval coproculture, it requires a trained taxonomist and is time consuming.^26^ Fortunately, the recent development of equine‐specific strongyle ITS2 nemabiome metabarcoding offers a promising approach for non‐invasive characterisation of mixed strongyle infections in horses,^15^ and researchers have begun using this approach in various parts of the world.^8^, ^15^, ^27^, ^28^, ^29^

Despite considerable research on equine strongyle diversity globally,^25^ the diversity of strongyles infecting horses in Western Asia, such as Iran, remains limited. While the exact population of equids in Iran is unknown, there are approximately 25,000 certified horses, along with athletic and working donkeys and mules distributed across the country.^30^ Most studies on strongyle infections in Iran have utilised traditional parasitological methods such as faecal egg counts and faecal cultures to report the prevalence of strongyle infections.^30^ Based on a limited number of post‐mortem studies using morphological identification of adult worms, 27 strongyle species across 10 genera have been recorded to date in this country.^30^, ^31^, ^32^ Given the diverse roles of horses in Iran, ranging from working animals to athletic companions, the varying levels of management and care they receive, as well as different geographical and climatic factors, it is important to study their strongyle parasite communities to develop effective parasite management practices.

The current study aimed to investigate the diversity of equine strongyles in northern Iran using both traditional morphological methods and nemabiome metabarcoding. By employing molecular techniques alongside conventional approaches, we aimed to increase knowledge of strongyle community composition in this region and to evaluate the effectiveness of different diagnostic methods. In particular, we aimed to expand the documented diversity of equine strongyle species in this understudied region and to generate new reference sequences for species absent from public databases, including potentially rare taxa not reported elsewhere. In addition, we aimed to determine if parasite communities differ across locations, and whether they were influenced by host age, sex or the diagnostic method used. This study offers novel insights into the diversity of strongyle parasites in northern Iran and contributes to the broader understanding of parasitic infections in horses.

## 2 MATERIALS AND METHODS

### 2.1 Sample collection

**FIGURE 1:**
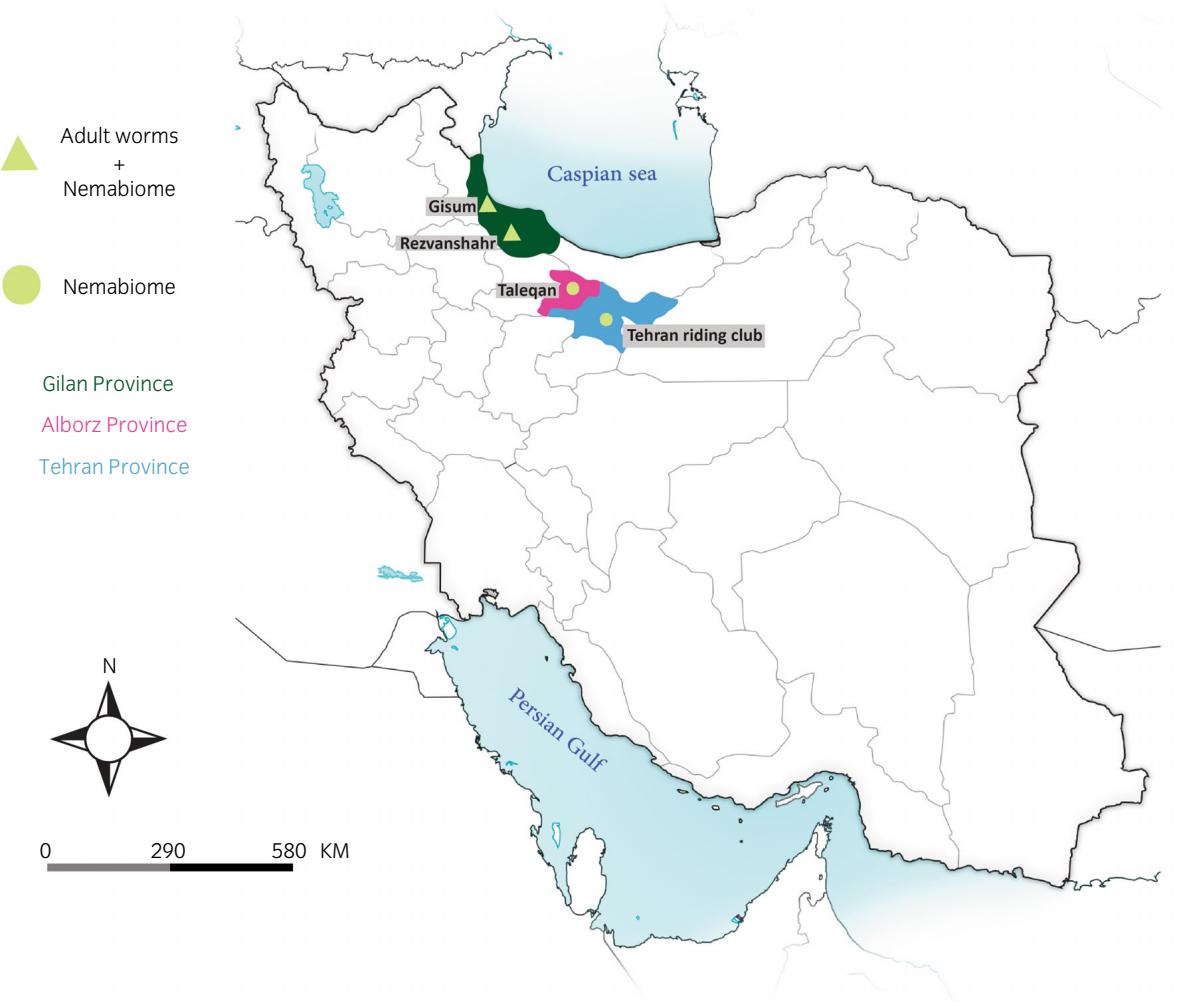
Map of Iran showing the location of provinces where horses were sampled. In Gilan Province (Rezvanshahr and Gisum farms), both morphological identification of adult worms and nemabiome metabarcoding were performed, while in Alborz and Tehran Provinces only nemabiome metabarcoding was conducted.

Faecal samples were collected from horses at four locations in three northern provinces of Iran (Gilan, Alborz and Tehran) between February and July 2022 (Figure 1, Table 1). At two farms in Gilan (Rezvanshahr and Gisum), faeces were obtained from both recently treated horses (24 h after ivermectin administration by horse owners, for recovery of adult parasites) and untreated horses (for larval cultures used in nemabiome metabarcoding). In total, 23 post‐treatment samples (18 from Rezvanshahr, 5 from Gisum) and 12 untreated samples (10 from Rezvanshahr, 2 from Gisum) were collected from these farms. Samples for two diagnostic methods (morphological identification of adult and nemabiome metabarcoding) originated from different horses but these were drawn from the same farms and thus subjected to the same management practices, grazing conditions, parasite transmission pressures and anthelmintic treatment regimes. Although pre‐ and post‐treatment samples from the same horses would have enabled direct within‐individual comparisons, the groups analysed by each diagnostic method represent independent subsets of the same underlying populations. At the remaining two locations—a farm in Alborz (Taleqan) and a riding club in Tehran—we only collected samples from untreated horses for nemabiome metabarcoding. At the Taleqan farm, four working horses residing in a fruit garden were sampled. At the Tehran riding club, 12 resident and 10 non‐resident horses were sampled. Resident horses had received anthelmintic treatment 4 months earlier, whereas the treatment history of all other horses included in the study was unknown. The age and sex of each horse were recorded based on information provided by owners.

**TABLE 1:** Summary of horse sampling by location, treatment status and number of samples collected.

Farm/location	Province	Sampling period	Post treatment (number of horses)	Faecal samples collected (number of horses)	Last anthelmintic treatment
Rezvanshahr	Guilan	Late February	18	10	Unknown
Gisum	Guilan	March	5	2	Unknown
Taleqan	Alborz	July	—	4	Unknown
Tehran (resident)	Tehran	March	—	12	4 months before sampling
Tehran (non‐resident)	Tehran	March	—	10	Unknown

### 2.2 Collection and morphological identification of adult Worms

For each of the 23 post‐treatment faecal samples, approximately 500 g were carefully examined to collect voided worms, resulting in a total of 1533 strongyle worms. Each worm was washed in saline buffer, and for some specimens, the posterior part was preserved in 70% ethanol for molecular analysis, while the anterior part was cleared using lactophenol (Sigma‐Aldrich, USA) for morphological identification. Other worms were identified morphologically without the clearing process.

Morphological identification was carried out using valid identification keys and based on the characteristics of the buccal capsule, mouth collar, cephalic papillae, internal leaf‐crown, external leaf‐crown, extra‐chitinous supports of the external leaf‐crown, and oesophageal funnel.^20^, ^33^

### 2.3 Faecal sample processing for larval culture and egg counts

To confirm strongyle infection, a faecal egg count (FEC) was performed on each of the faecal samples designated for nemabiome metabarcoding using a modified McMaster technique as previously described.^34^ Briefly, 4 g of faeces were first homogenised in 26 mL of Sheather's sugar solution (specific gravity of 1.27). The mixture was then filtered, thoroughly mixed and aliquoted into two 0.15 mL chambers of a McMaster slide. The number of strongyle eggs was counted under a microscope, and eggs per gram (EPG) of faeces were calculated by summing counts from both chambers and multiplying by 25.

Of the 38 samples originally collected, 25 tested positive for strongyle infection and were submitted to larval culture. These included 10 from Rezvanshahr, 2 from Gisum, 4 from Taleqan and 9 from the Tehran riding club (2 resident and 7 non‐resident horses). Coprocultures were prepared by placing 30–50 g of faeces in a glass cup, misting with tap water, covering with a plastic petri dish, and incubating at room temperature (~22°C) for 14 days. Samples were kept moist by spraying with tap water every other day. Following incubation, L3 larvae were extracted using the glass over petri dish method^35^ for 8–10 h, then fixed in 70% ethanol and stored at −20°C until further analysis.

### 2.4 DNA preparation from larval coprocultures

The number of cultured L3 larvae obtained from coprocultures was estimated based on two counts of 5 μL aliquots; then DNA lysates were prepared from aliquots of ~2500 L3s using a protocol adapted from Poissant et al. (2021).^15^ Specifically, ethanol was removed using a vacuum evaporator rather than multiple rounds of washing, and cells were disrupted using bead beating rather than prolonged incubation. Briefly, excess ethanol was first removed following centrifugation at 13,000 g for 4 min. Remaining ethanol was then removed using a vacuum evaporator set at room temperature for ~2 h and larvae were re‐suspended in 150 μL of lysis buffer. Two 5 mm stainless steel beads (Qiagen, Germany) and 6 μL of Proteinase K (20 mg/mL) were added to each sample, which were then placed in a Vortex‐Genie 2 fitted with an adapter (Qiagen, Germany) for 10 min of bead beating. Samples were then placed on a plate shaker (300 rpm) in an incubator set to 55°C for 2 h. Following lysis, proteinase K was inactivated by incubation at 95°C for 20 min. Finally, 1:10 dilutions of the DNA lysate were prepared with molecular‐grade water and stored at −80°C until further processing.

### 2.5 ITS2 PCR amplification and sequencing of strongyle larvae

Following previously published protocols,^12^ the ITS2 gene region was amplified using the NC1 and NC2 primers^36^ with up to three random bases preceding the priming sequence and an adapter sequence to allow incorporation of barcodes in a subsequent PCR. The 25 μL PCR reaction included: 5 μL of KAPA HiFi Buffer (Roche, Switzerland), 0.75 μL of each primer (10 μM), 0.75 μL of dNTPs (10 mM), 0.5 μL KAPA HiFi Polymerase (0.5 U), 5 μL of DNA template (1:10 diluted DNA) and 12.5 μL of molecular grade water. The initial PCR was conducted with an initial denaturation at 95°C for 3 min, followed by 25 cycles of 98°C for 20 s, 62°C for 15 s and 72°C for 15 s, with a final extension at 72°C for 2 min. The PCR products were purified using AMPure XP magnetic beads (Beckman Coulter, USA). A second PCR was then performed on the purified products to incorporate IDT for Illumina unique dual indices (UDI) and Illumina sequencing adaptors, allowing for sample‐specific tagging and minimising index hopping. The thermocycling condition of the second PCR consisted of 98°C for 45 s, followed by 7 cycles of 98°C for 20 s, 63°C for 20 s and a final extension at 72°C for 2 min. Following magnetic bead purification to ensure high‐quality libraries for sequencing, the DNA concentration of each sample was quantified using a BioTek Take3 microplate reader, and 100 ng of DNA from each sample was then pooled in a single library. This library was quantified using a Qubit 4 Fluorometer (Thermo Fisher Scientific, USA), and sequenced on an Illumina MiSeq platform with a MiSeq Reagent Kit v2 Nano (Illumina, USA) using a 12 pM dilution with 20% PhiX spike‐in.

### 2.6 Addition of a *Triodontophorus tenuicollis* ITS1‐5.8S‐ITS2 sequence to public databases

**FIGURE 2:**
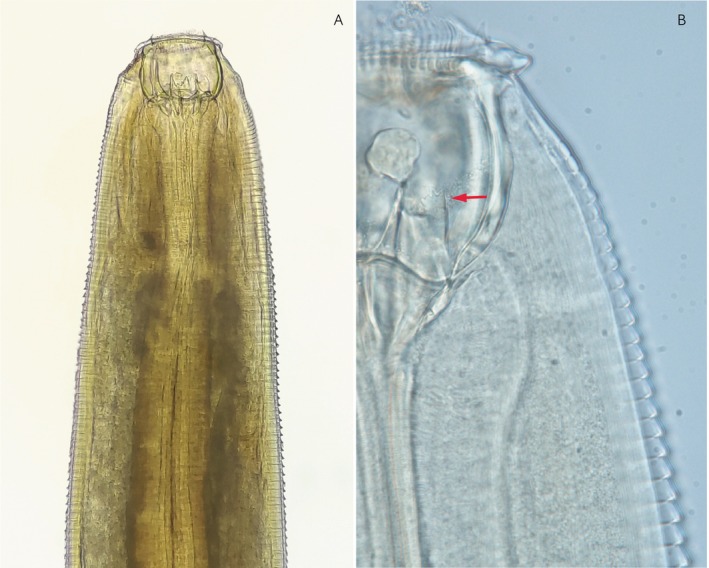
Triodontophorus tenuicollis adult worm. A. Freshly collected specimen. Anterior section and the buccal capsule. B. Cleared specimen. Serrated cuticle and the denticulated tooth (arrow).

Morphological identification of adult worms from post‐treatment samples recovered four specimens of *Triodontophorus tenuicollis*, a non‐migratory large strongyle (Figure 2). At the time of the study, no representative sequence for *T. tenuicollis* was available in public databases.^37^ Although Diekmann et al. (2025) recently uploaded ITS2 sequences of this species into GenBank,^38^ these were not publicly accessible at the time of analysis. Consequently, we generated an ITS1‐5.8S‐ITS2 sequence for this species in order to allow its detection in this and other nemabiome analyses.

DNA was extracted from the abdominal section of a randomly selected *T. tenuicollis* specimen using the DNeasy Blood & Tissue Kit (Qiagen, Germany) according to the manufacturer's instructions, and the DNA concentration was assessed using a Qubit 4 Fluorometer. PCR amplification of the ITS1‐5.8S‐ITS2 region was done using the NC5 (5′‐GTA GGT GAA CCT GCG GAA GGA TCA TT‐3′) and NC2 (5′‐TTA GTT TCT TTT CCT CCG CT‐3′) primers previously described for nematodes.^39^ The 25 μL PCR reaction included: 5 μL of KAPA HiFi Buffer (Roche, Switzerland), 0.75 μL of each primer (10 μM), 0.75 μL of dNTPs (10 mM), 0.5 μL KAPA HiFi Polymerase (0.5 U), 15 μL of DNA template (approximately 10 ng of DNA) and 2.25 μL of molecular‐grade water. Amplification followed the cycling protocol: initial denaturation at 95°C for 2 min, followed by 35 cycles of denaturation at 98°C for 20 s, annealing at 62°C for 15 s and extension at 72°C for 15 s, with a final extension at 72°C for 2 min. The amplified products were electrophoresed on 2% agarose gels, visualised using a UV transilluminator and excised from the gel for purification using the QIAquick Gel Extraction Kit (Qiagen, Germany) following manufacturer protocols. Purified DNA was then sequenced using the Sanger method for each primer using an Applied Biosystems™ 3730xl DNA Analyser (USA) at the University of Calgary Centre for Health Genomics and Informatics (CHGI). Sequences obtained from CHGI were inspected and manually edited, and forward and reverse reads were merged using the UniPro UGENE software v42.^40^ Using the NCBI BLAST tool (available at https://blast.ncbi.nlm.nih.gov/Blast.cgi), we assessed the sequence identity of the newly sequenced species against closely related species to confirm their molecular identity.

### 2.7 Nemabiome bioinformatic analyses

The raw output from the Miseq sequencing run was processed following an updated version (v2) of the bioinformatics pipeline published by Poissant et al. (2021) (available at https://data.mendeley.com/datasets/vhyysw8xt2/2) with up to 2.5% base mismatches allowed during the merging of forward and reverse reads.^41^ For taxonomic assignments, the ITS2 reference database v1.6.0^37^ accessed at https://www.nemabiome.ca was curated following steps in Poissant et al. (2021), and the sequence of *T. tenuicolli*s was manually added. Taxonomic classification was performed using the DADA2 *assignTaxonomy* function, with an assignment confidence threshold of 80% or higher. Classified ASVs were then consolidated at the species level. To account for the fact that *Strongylus* species generally yield more amplicons per larva than other equine strongyles,^15^ the number of reads of three *Strongylus* species was divided by a correction factor derived by Ahn et al. (2024) (*S. edendatus*: 1.52, *S. equinus*: 1.81, *S. vulgaris*: 4.66).^42^ Following correction, the relative abundance of each species was calculated by dividing the number of species‐specific reads by the total number of reads per sample. Finally, to account for possible index hopping, contaminations and sequencing errors, species constituting less than 1/2500 of the total reads in a sample were excluded from the analysis.

### 2.8 Community and statistical analyses

To assess variation in nematode community composition across samples, we computed Jaccard and Bray–Curtis dissimilarity indices using presence/absence and relative abundance data, respectively, for both the molecular and morphologically identified samples. Morphologically identified samples with less than 25 worms were excluded from community composition analyses to minimise noise from poorly resolved communities (see Table S1 for the list of excluded samples). Principal Coordinates Analysis (PCoA) was then applied to the dissimilarity matrices to visualise variation among samples. These analyses were performed using the R package *vegan* 2.6–4.^43^

A permutational multivariate analysis of variance (PERMANOVA) was also performed on the dissimilarity matrices using *adonis2* with 9999 permutations from the R package *vegan* ^43^ to assess the influence of population (with resident and non‐resident horses of the riding club treated as separate populations), method (nemabiome or morphology), age and sex (male, female) on the composition of strongyle communities. We also used generalised linear models (GLMs) with Poisson error using the package *glmmTMB* ^44^ to test the effect of the same variables on species richness. Finally, we analysed Shannon diversity as a response variable using a GLM with a Gaussian error distribution, assessing the effect of the same predictor variables. Pairwise comparisons between populations and methods of identification were performed using estimated marginal means using the package *emmeans* ^45^ based on the fitted model. To evaluate whether including method as a variable might have inadvertently influenced results for other variables, given that only two of the five populations were analysed using both methods, we also ran method‐specific models. These models yielded conclusions consistent with those of the full model including method as a predictor. Accordingly, we only present the results of the full model.

## 3 RESULTS

### 3.1 Morphological findings

**FIGURE 3:**
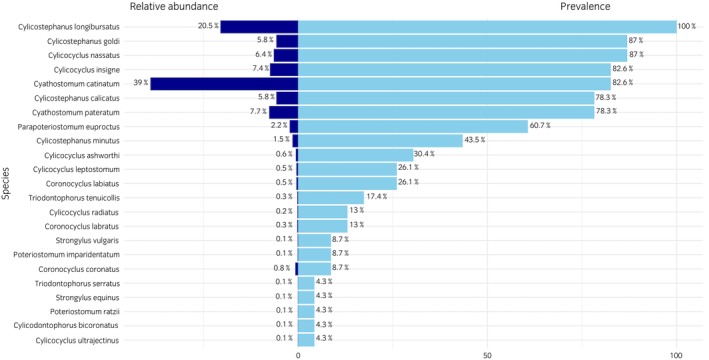
Relative abundance (%) and prevalence (%) of parasitic strongyle species in 23 horses from two farms in Gilan Province, northern Iran, based on morphological identification of adult worms collected from post‐anthelmintic treatment faecal samples.

**FIGURE 4:**
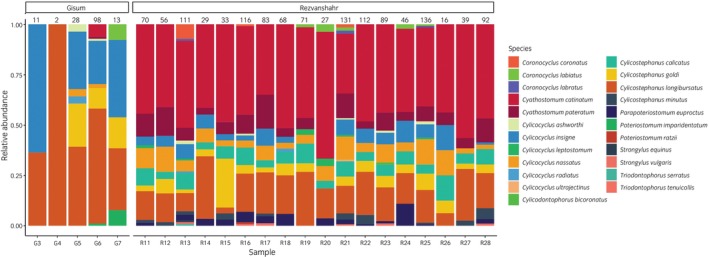
Relative abundance of parasitic strongyle species in 23 horses from 2 farms (Gisum and Rezvanhahr) in Gilan province, northern Iran, based on morphological identification of adult worms collected from post‐anthelminthic treatment faecal samples. The total number of worms identified is indicated above each bar.

In total, 1476 of the 1533 strongyle worms collected were adults and morphologically identified, while 57 were fourth‐stage larvae and not identified (Table 2, Figures 3 and 4). The number of strongyles identified from each faecal sample ranged between 2 and 142. In total, 23 strongyle species from 9 genera were recorded. The most prevalent species were *Cylicostephanus longibursatus, Cylicostephanus goldi, Cylicocyclus nassatus, Cyathostomum catinatum* and *Cylicocyclus insigne* with 100%, 87%, 87%, 82.6% and 82.6%, respectively.

**TABLE 2:** Prevalence of parasitic strongyle species in 23 horses from Gilan Province in northern Iran based on morphological identification of 1476 adult worm specimens collected from post‐anthelminthic treatment faecal samples.

Species	Number	Prevalence %	Intensity		Relative abundance %
			Mean	Range	
*Coronocyclus coronatus*	11	8.7	3.66	1–8	0.7
*Coronocyclus labiatus*	7	26.1	1.16	1–2	0.5
*Coronocyclus labratus*	4	13	1.33	1–2	0.3
*Cyathostomum catinatum*	576	82.6	30.31	6–54	39.0
*Cyathostomum pateratum*	113	78.3	6.27	1–16	7.6
*Cylicocyclus ashworthi*	9	30.4	1.28	1–2	0.6
*Cylicocyclus insigne*	110	82.6	5.78	1–21	7.4
*Cylicocyclus leptostomum*	7	26.1	1.16	1–2	0.5
*Cylicocyclus nassatus*	95	87	4.75	1–17	6.4
*Cylicocyclus radiatus*	3	13	1	1–1	0.2
*Cylicocyclus ultrajectinus*	1	4.3	1	—	0.1
*Cylicodontophorus bicoronatus*	1	4.3	1	—	0.1
*Cylicostephanus calicatus*	85	78.3	4.72	1–10	5.8
*Cylicostephanus goldi*	85	87	4.25	1–11	5.8
*Cylicostephanus longibursatus*	303	100	13.17	1–56	20.5
*Cylicostephanus minutus*	22	43.5	2.2	1–5	1.5
*Parapoteriostomum euproctus*	33	60.7	2.35	1–6	2.2
*Poteriostomum imparidentatum*	2	8.7	1	1–1	0.1
*Poteriostomum ratzii*	1	4.3	1	—	0.1
*Strongylus equinus*	1	4.3	1	—	0.1
*Strongylus vulgaris*	2	8.7	1	—	0.1
*Triodontophorus tenuicollis*	4	17.4	1	—	0.3
*Triodontophorus serratus*	1	4.3	1	—	0.1

*Note*: Intensity refers to the number of worms in each positive sample. Relative abundance was calculated as the proportion of each species compared to the total number of worms across all samples.

### 3.2 Faecal egg counts

Overall, faecal egg counts ranged from 0 to 1025 EPG (Table 3). Horses from the Rezvanshahr, Gisum and Taleqan farms had mean EPG of 672 ± 283 (range: 300–1025), 275 ± 25 (range: 250–300) and 250 ± 106 (range: 125–350), respectively. In contrast, strongyle infection was less prevalent among horses sampled at the horse‐riding club in Tehran, with infection rates of 16.7% and 70% for resident and non‐resident horses, respectively. The resident horses had received anthelmintic treatment with ivermectin 4 months earlier, which likely contributed to the lower prevalence observed in this group. EPG also appeared to be comparatively lower in Tehran horses, with mean values of 16.7 ± 44.5 (range: 0–250) and 62.5 ± 76 (range: 0–150) for resident and non‐residents, respectively.

**TABLE 3:** Strongyle infection prevalence and eggs per gram of faeces (EPG) in non‐treated horses from four locations in northern Iran.

Location	Number	Horse age mean and range	Strongyle prevalence	Strongyle EPG mean ± SD	Strongyle EPG range
Rezvanshahr farm	10	5.3 (2–11)	100%	672 ± 283	300–1025
Gisum farm	2	6.5 (6–7)	100%	275 ± 25	250–300
Taleqan farm	4	6.5 (2–11)	100%	250 ± 106	125–350
Tehran resident	12 (2)	7.1 (1–16)	16.7%	16.7 ± 44.5	0–250
Tehran non‐resident	10 (7)	8.3 (2–24)	70%	62.5 ± 76	0–150

*Note*: Horses sampled at a Tehran riding club were separated into resident and non‐resident populations for analysis. The number of horses sampled per site and horse age (mean and range) are also presented.

### 3.3 *T. tenuicollis* ITS1‐5.8S‐ITS2 sequence

An 832 bp ITS1‐5.8s‐ITS2 sequence was successfully sequenced for an adult *T. tenuicollis* specimen. The sequence was found to have 97.81% identity with *Triodontophorus nipponicus* (KP693437.1).

### 3.4 Nemabiome metabarcoding analysis

**FIGURE 5:**
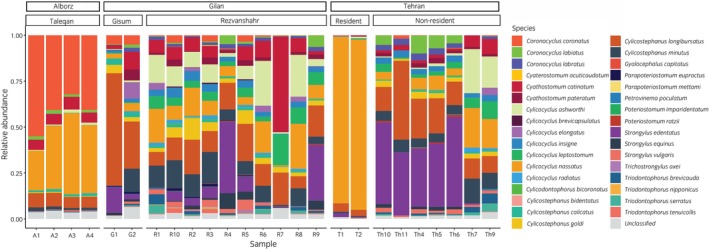
Relative abundance of strongyle species in 25 horses from three farms from Alborz and Gilan provinces and a riding club from Tehran in northern Iran based on nemabiome metabarcoding applied to larval cultures.

The number of paired reads generated by the Illumina MiSeq ranged from 17,814 to 40,589 per sample. Following quality filtering, 16,296 to 37,797 paired reads remained per sample. Details about the number of reads/amplicons generated and those remaining after bioinformatic processing are summarised in Table S2. Of the 282 amplicon sequence variants (ASVs) identified, 252 (89.4%) were assigned to species with at least 80% bootstrap support. In total, 97.6% of the retained sequencing reads (585,931/600,319) were assigned to species. Overall, 33 species belonging to 13 genera were detected including 32 equine strongyles as well as *T. axei* (Figure 5).

At the two farms from Gilan Province, totals of 24 and 30 species were identified using nemabiome metabarcoding (Figure 5). In Rezvanshahr, the prevalence of *S. vulgaris, S. edentatus* and *S. equinus* was 100%, 80% and 80%, respectively, whereas all of these species were observed in both horses from Gisum. Furthermore, 24 species of small strongyles had a prevalence of ≤50% in Rezvanshahr and Gisum (Table S3), while *T. axei* was only detected in four horses from Rezvanshahr.

At the Taleqan farm location in the Alborz Province, 14 species of strongyles were detected in the 4 horses examined, with a largely similar species composition across individuals (Figure 5). The only species that were not detected in all four horses were *Cylicocyclus leptostomum* and *Cylicocyclus radiatus*, which were only found in three and one horses, respectively.

At the Tehran riding club, totals of 14 and 31 species were observed in resident and non‐resident horses, respectively. Among resident horses, *S. vulgaris* and *S. edentatus* were found in 50% and 100% of horses, while *S. equinus* was not detected (Table S3, Figure 5). In non‐resident horses, the prevalences of these species were 100%, 57.1% and 100%, respectively. The relative abundance *of S. vulgaris* and *S. edentatus* was notably lower in resident compared to non‐resident horses (Figure 5). Distinct groups of non‐resident horses with contrasting parasite communities were also apparent (Figure 5).

### 3.5 Community analysis

**FIGURE 6:**
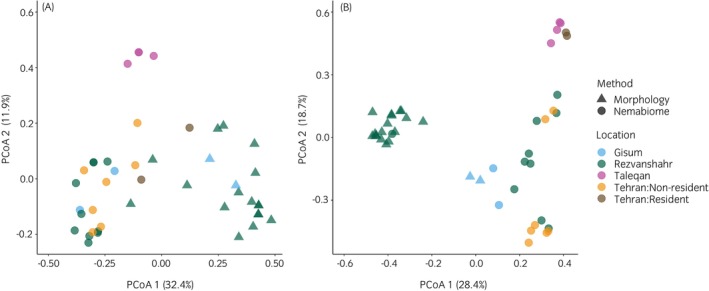
Principal coordinate analysis of the Jaccard (A) and Bray‐Curtis (B) dissimilarity matrices based on presence/absence and relative abundance of strongyle species among 44 horses in northern Iran. The percentages show the variance explained by principal coordinate axes. Communities were measured using either morphological identification of adults or nemabiome metabarcoding of cultured L3 larvae. Four samples with fewer than 25 adult worms identified were omitted from the analysis to avoid introducing noise from poorly resolved communities.

The PCoA plots based on Jaccard (Figure 6A) and Bray‐Curtis (Figure 6B) indices suggested that strongyle communities clustered by location and identification method. On the Jaccard plot, while some samples from Rezvanshahr, Gisum and Tehran overlapped, those from Taleqan showed distinct clustering (Figure 6A). The Bray‐Curtis‐based PCoA, which accounts for relative abundances, revealed clearer separation of populations (Figure 6B). In that case, Taleqan and Tehran resident horses formed distinct clusters, while Rezvanshahr and Gisum overlapped with Tehran non‐resident horses to a lesser extent. Regarding the methods of identification and quantification, nemabiome‐ and morphology‐based identification appeared to yield different community structures for where they were both applied (Rezvanshahr and Gisum). However, distinctiveness appeared to be influenced by the dissimilarity index used, with clustering by method appearing to be more pronounced with Jaccard for Gisum, and with Bray‐Curtis for Rezvanshahr (Figure 6). Four morphologically identified samples with fewer than 25 worms were excluded from these analyses to avoid biases from poorly resolved communities.

The PERMANOVA revealed that community composition was significantly explained by population (Jaccard: *R* ^2^ = 0.216, *p* < 0.001, Bray‐Curtis: *R* ^2^ = 0.361, *p* < 0.001), and method (Jaccard: *R* ^2^ = 0.299, *p* < 0.001, Bray‐Curtis: *R* ^2^ = 0.177, *p* < 0.001), but did not detect significant effects of host sex (Jaccard: *R* ^2^ = 0.016, *p* = 0.176, Bray‐Curtis: *R* ^2^ = 0.008, *p* = 0.418) or age (Jaccard: *R* ^2^ = 0.005, *p* = 0.860, Bray‐Curtis: *R* ^2^ = 0.003, *p* = 0.858).

**FIGURE 7:**
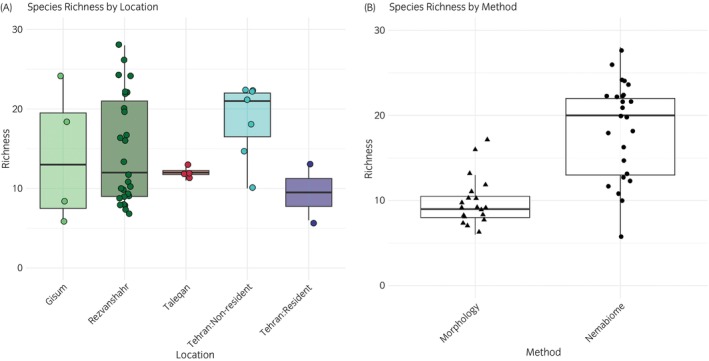
Observed species richness across different farms (A) and methodological approaches (B). The plots are based on raw data.

**FIGURE 8:**
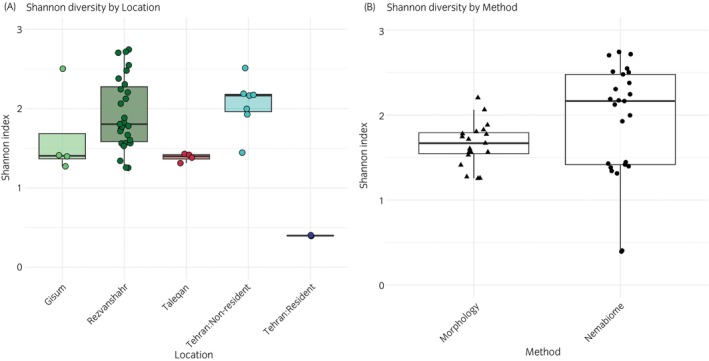
Observed Shannon diversity index across different farms (A) and methodological approaches (B). The plots are based on raw data.

Both species richness and Shannon diversity were significantly associated with population and method of identification (all *p* < 0.001; Table 4). In contrast, there was no significant effect of host age (Estimate = 0.009 ± 0.009 for richness, 0.010 ± 0.011 for diversity; all *p* > 0.3) or between sexes (with males as the reference: Estimate = −0.095 ± 0.085 for richness, −0.153 ± 0.091 for diversity; all *p* > 0.09) for either metric. Pairwise comparisons of species richness and Shannon diversity by locations and methods are summarised in Table S4. Species richness varied significantly between populations, with some horses with unknown treatment history showing higher diversity than others (Table S4). For instance, resident horses at the Tehran riding club had consistently lower richness and diversity compared to non‐resident horses and the three sampled farms (Figures 7 and 8). Taleqan horses also showed lower diversity and richness compared to Rezvanshahr horses, while there were no significant differences observed between Taleqan and Gisum (Figures 7 and 8). In terms of the method of identification, the nemabiome method consistently detected greater parasite diversity compared to morphological approaches (*p* < 0.001) (Figure 7, Table S4).

**TABLE 4:** ANOVA results for generalised linear models of species richness and Shannon diversity.

Model	Factor	Chi‐Square	Df	*p‐*value
Species richness	Farm	28.290	4	**< 0.001**
	Method	72.324	1	**< 0.001**
	Age	0.881	1	0.347
	Sex	1.239	1	0.265
Shannon diversity	Farm	103.661	4	**< 0.001**
	Method	38.653	1	**< 0.001**
	Age	0.698	1	0.403
	Sex	2.775	1	0.095

*Note*: Significance was assessed at *p* < 0.05, with significant values shown in bold.

## 4 DISCUSSION

This study characterised equine strongyle communities from four different locations in northern Iran including three farms and both resident and non‐resident horses at a riding club. Morphological and nemabiome metabarcoding methods detected a total of 33 species of equine strongyles. Morphological identification of adult worms detected 23 species of strongyles, while nemabiome metabarcoding detected 32 as well as *T. axei*. Some of these horses, particularly from farms in the Gilan Province, harboured a high diversity of strongyles, with richness levels comparable to those reported in unmanaged feral populations.^15^, ^42^ In contrast, resident horses from the Tehran riding club exhibited lower diversity, consistent with patterns typically observed in other managed domestic horse populations.^28^, ^29^, ^46^, ^47^

We report a higher diversity of equine strongyle species in Iran compared to previous reports which indicated the presence of 27 species based on morphological identification.^30^ While we did not detect five previously reported species, including *Cyathostomum alveatum, Cyathostomum tetracanthum, Cylicocyclus auriculatus, Cylicostephanus hybridus* and *Oesophagodontus robustus*, we report 11 new ones for this area: *T. nipponicus, Cra. acuticaudatum, Cyc. leptostomum, Cyc. ashworthi, Cyc. ultrajectinus, Cylicocyclus brevicapsulatus, Cylicostephanus bidenatus, Par. euproctus, Parapoteriostomum mettami, Pot. ratzii* and *Petrovinema poculatum*. Among these species, *Cyc. ashworti* and *Cyc. leptostomum* are common in domestic horses, whereas the others are considered rare.^25^ Species considered rare tended to have low relative abundance, such as *Cyc. ultrajectinus* which was only detected in a single individual. However, other rare species such as *P. euproctus* had a high prevalence in our study.

Identification method played a significant role in explaining strongyle diversity, with nemabiome metabarcoding noticeably detecting more species than morphological identification. For example, nemabiome metabarcoding detected 31 strongyle species in Rezvanshahr, while morphological identification only detected 23. Nemabiome metabarcoding generally detected more rare species that had low relative abundance. This could be due to differences in the number of specimens that were processed between methods, with nemabiome metabarcoding sequencing ~2500 L3s per sample, while morphological identification was limited by the number of adult worms obtained. With many of the rare species having relative abundances lower than 0.1, a larger number of adult worms would likely be required to detect all the species identified using nemabiome metabarcoding. While previous reports suggested that 1300 adult worms are generally sufficient to describe the composition of equine strongyle communities,^48^ our findings suggest that this threshold might still miss some rare species, particularly under field conditions. In addition to sensitivity issues, it is worth highlighting that obtaining specimens for morphological identification without sacrificing horses requires anthelmintic administration. Since anthelmintic resistance is species‐specific^8^ and the community of adult worms expelled can depend on when samples are collected following treatment,^49^ such methods may lead to biased composition estimates. For instance, only a single post‐treatment time point was examined, which may have limited the recovery of some species.

We acknowledge that our results are limited by the absence of paired samples, which would have enabled direct within‐individual comparison between morphological and molecular characterisations of strongyle communities. Nonetheless, comparison of independent subsets of individuals from the same farms provided an adequate basis for comparing the two diagnostic methods. Given the time, expertise and resources required for morphological identification, nemabiome metabarcoding provides a more accessible and scalable alternative for monitoring infections, particularly in regions with high parasite diversity and limited taxonomic capacity. Moreover, it is understandable that adult worm recovery following anthelmintic treatment and larval identification from untreated coprocultures may lead to different results. For example, outcomes can be influenced by the life history of individual strongyle species, including variation in fecundity relative to other species. In addition, molecular methods are subject to biological or technical biases such as copy number variation among species, primer bias and differences in the number of amplicons generated per larva.^15^, ^42^ Therefore, our findings provide practical insights into the strengths, limitations and complementarity of both approaches for studying the equine strongyles.

Nemabiome metabarcoding was particularly important for the detection of highly pathogenic species of large strongyles. Morphological identification detected *S. vulgaris* in 8.7% of samples, similar to a previous study in the northwest of Iran which showed a prevalence of 6.5%.^50^ In contrast, our nemabiome analysis detected *S. vulgaris* in 96% of studied horses, similar to patterns observed in unmanaged feral horse populations.^42^, ^51^ This difference indicates that *S. vulgaris* is likely more common in northern Iran than initially thought, and that current parasite management practices in the region may not be sufficient to control for highly pathogenic species. However, an important consideration is that nemabiome metabarcoding characterises communities based on egg shedding, while morphological identification quantifies adult worm burden. Crucially, adult worm burden and egg shedding are not strongly correlated, and egg shedding emphasises transmission potential rather than the physical impact of parasites in a horse.^52^ Furthermore, large migratory strongyle are not detectable during their prepatent (and most pathogenic) period using either adults collected post‐treatment or nemabiome metabarcoding.^53^ In that regard, it is crucial that researchers and clinicians choose a diagnostic method that is most appropriate to address their objectives. Given that nemabiome metabarcoding can detect more species, requires limited training, and is less dependent on sample collection, we suggest that nemabiome metabarcoding is particularly valuable as a monitoring tool for parasite transmission.

Resident horses in Tehran had lower species richness and lower relative abundance of *Strongylus* species compared to non‐resident horses. These resident horses were known to have received anthelmintic treatment (ivermectin) 4 months prior to treatment, which likely disrupted strongyle community composition, particularly species such as *S. vulgaris* with longer prepatent periods. Differences in parasite management between horse owners may explain the contrasting patterns observed, with resident horses likely receiving more regular veterinary care than the non‐resident horses. However, it is important to note that only two resident horses met the inclusion criteria for nemabiome analysis, and thus, these findings should be interpreted with caution.

While nemabiome metabarcoding generally detected more species, its accuracy is contingent on a robust reference database, as seen with *T. tenuicollis* which was initially only identified using morphological methods and only detected with nemabiome metabarcoding when its ITS2 sequence was manually added to the reference database. Furthermore, many species, particularly those that are rare, are still missing from ITS2 reference databases and therefore remain undetectable using current nemabiome approaches (e.g., *Cylicostephanus hybridus*, *Cyathostomum alveatum, Skrjabinodentus longiconus, Triodontophorus minor* and *Caballonema longicapsulatum*). It is also important to note that coproculture may introduce its own biases, as some species may not survive or develop efficiently under certain culture conditions,^54^ potentially underrepresenting their true abundance in the original sample.^12^ In addition, evidence suggests that some genetic markers, including the ITS2 region, lack the resolution needed to distinguish closely related equine strongyle species.^55^ As a result, nemabiome metabarcoding may be unable to confidently identify certain species, even when they are represented in reference databases.

The morphological identification and subsequent sequencing of *T. tenuicollis* enabled the identification of this species using nemabiome metabarcoding. However, when *T. tenuicollis* was excluded from the reference database, some ASVs were instead assigned to another species (*T. nipponicus*) with high confidence, demonstrating the risk of false species identification due to incomplete reference databases. A recent study showed that the morphological identification and subsequent inclusion of the ITS2 sequence of *Hsiungia pekingensis*, an extremely rare strongyle species, facilitated its reliable detection and expanded its known geographic range through nemabiome sequencing.^56^ However, the ASVs representing *H. pekingensis* were distinct enough that, even in the absence of its sequence in the ITS2 database, they were not misassigned to other species (i.e., low confidence in taxonomic assignment). These findings emphasise the continued importance of morphological validation to confirm species presence and refine molecular diagnostic methods. Indeed, while nemabiome metabarcoding appears to be more sensitive than traditional approaches, morphological studies remain essential to prevent misidentification and improve the accuracy of DNA‐based diagnostics.^57^

A key finding of this study is that strongyle community composition varies among sampling locations. The influence of herd and farm differences on parasite community composition has been documented in other studies as well. For example, nemabiome metabarcoding demonstrated distinct differences in nematode species composition between conservation and commercial bison herds.^58^ It has also been shown that social grouping and shared habitats can contribute to similarities in parasite communities among feral horses.^42^ In contrast, a study in Thailand that sampled horses from five different regions found no significant differences in strongylid richness and diversity,^28^ suggesting that such patterns may be context‐dependent and influenced by local factors and study design. Species richness and diversity were notably higher in horses from Gisum and Rezvanshahr compared to resident horses of Tehran and Taleqan, highlighting the possible influence of local practices. Differences in practices are also likely responsible for the detection of *T. axei* in some of our nemabiome samples, similar to a previous study that detected *T. axei* and other sheep nematodes in a population of unmanaged ponies in Scotland and attributed its presence to co‐grazing with sheep.^29^ In our study, the horses in which *T. axei* was detected were also co‐grazing with cattle, further supporting the role of mixed‐grazing in facilitating cross‐species parasite transmission. While *T. axei* is not known to be pathogenic in horses,^59^ multi‐species farms may require unique parasite management plans if host‐sharing is a concern. Given that horses appear to reflect infection patterns based on their farm, and that drug resistance can be farm‐ and species‐specific,^8^ vigilance may be required if horses are being transferred between locations.

Neither host sex nor age had a significant effect on species composition, richness or diversity, suggesting that environmental‐ and management‐related factors may play more dominant roles in structuring strongyle communities in the studied population. This finding contrasts with previous studies in horses where age‐related differences in strongyle infection have been reported,^27^, ^60^, ^61^ indicating that host factors may be context‐dependent and influenced by regional or farm‐specific conditions. However, a key limitation of this study was the reliance on data provided by horse owners which may have been inaccurate, including information on age and sex. Additionally, the inability to inquire about the origin of the Tehran non‐resident horses posed another limitation. In particular, the observed high prevalence of large strongyles and rare species in non‐resident horses of Tehran may have been influenced by previous anthelmintic management practices, but this could not be directly assessed.

## 5 CONCLUSION

This study offers the most comprehensive assessment of equine strongyle diversity in northern Iran conducted to date, identifying 33 species, including 11 newly recorded ones. While this study was limited in scope and should not be interpreted as a nationally representative survey, the diversity documented here provides valuable insights into understudied strongyle communities in this region and highlights the importance of future work to build a more comprehensive national picture. Nemabiome metabarcoding detected more species than morphological methods, highlighting its sensitivity in parasite identification. The nemabiome approach was particularly effective in detecting the highly pathogenic *S. vulgaris*, which may otherwise be overlooked. However, traditional morphological techniques remain vital for validating and refining molecular identifications, ensuring accuracy in species detection. The high diversity of strongyle species observed across different farms and between resident and non‐resident horses underscores potential gaps in current parasite management practices in the region. In particular, site‐specific practices and differences in veterinary care and management strategies are likely to shape infection patterns. Our findings support integrated parasite control strategies that combine both molecular and morphological approaches. Enhanced surveillance, continuous improvements in genetic reference databases, and tailored farm management practices are essential to accurately monitor strongyle communities and mitigate the impact of these infections on equine health in Iran and beyond.

## FUNDING INFORMATION

This project was funded by grants from the Margaret Gunn Endowment for Animal Health Research, the University of Calgary Faculty of Veterinary Medicine Clinical Research Fund, and the Zoetis Investment in Innovation Fund to J. P., as well as Alberta Graduate Excellence and the University of Calgary Provost's Doctoral scholarships to S. M.

## CONFLICT OF INTEREST STATEMENT

The authors declare no conflicts of interest.

## AUTHOR CONTRIBUTIONS

**Sina Mohtasebi:** Investigation; writing – original draft; writing – review and editing; visualization; methodology; formal analysis; data curation; conceptualization. **Sangwook Ahn:** Investigation; writing – review and editing; formal analysis; methodology; software. **Mahan Karimi:** Investigation; writing – review and editing. **Mohammad Saberi:** Investigation; writing – review and editing. **John S. Gilleard:** Writing – review and editing; supervision. **Jocelyn Poissant:** Conceptualization; writing – review and editing; project administration; funding acquisition; supervision.

## DATA INTEGRITY STATEMENT

Sina Mohtasebi had full access to all the data in the study and takes responsibility for the integrity of the data and the accuracy of the data analysis.

## ETHICAL ANIMAL RESEARCH

This study was conducted under the University of Calgary Veterinary Sciences Animal Care Committee Animal Use Protocol AC22‐0030.

## INFORMED CONSENT

Consent was obtained from the horse owners and the club for the publication of data from the samples obtained from their properties.

## Supporting information


**Table S1:** Post‐anthelmintic treatment samples with less than 25 adult worms recovered that were excluded from the analysis.

**Table S2:** The number of raw pairs of reads generated by the Illumina MiSeq and the number left at each stage of the metabarcoding bioinformatics pipeline for pools of ~2500 L3s isolated from faeces of horses in Gisum (G), Taleqan (A), Rezvanshahr (R), Tehran resident (T) and Tehran non‐resident (Th). The percentage of retained amplicons assigned to a species with ≥80 bootstrap support, and the number of species identified, are also presented.

**Table S3:** Prevalence of 32 strongyle species identified using nemabiome metabarcoding for 25 faecal samples from four locations and including both resident and non‐resident horses at a horse‐riding club in Tehran, Iran.

**Table S4:** Pairwise comparisons of species richness and Shannon diversity by locations and methods, estimated using estimated marginal means. The direction of each estimate is relative to the first level in the pair. Bolded values indicate statistically significant comparisons.

## Data Availability

The *T. tenuicollis* ITS1‐5.8S‐ITS2 sequence generated in this study has been added to GenBank (accession number PX597371) and the raw nemabiome ITS2 sequencing data for the pre‐treatment samples have been uploaded to the NCBI Sequence Read Archive (SRA; project PRJNA1356949).
