# Supplementary material for: Enhanced detection of equine strongyles: Insights from morphological and nemabiome metabarcoding approaches in northern Iran
Source: Equine Vet J. 2025 Nov 29;58(2):508–22. doi: 10.1111/evj.70120 (PMC12892384; doi:10.1111/evj.70120)
Supplement: Supplementary file 1 — Table S1: Post‐anthelmintic treatment samples with less than 25 adult worms recovered that were excluded from the analysis. [file EVJ-58-508-s003.pdf]

Equine Veterinary Journal BEVA

Table S1: Post-anthelmintic treatment samples with less than 25 adult worms recovered that were excluded from the analysis.

Samples with less than 25 worms	Farm	Number of worms
**G3**	Gisum	11
**G4**	Gisum	2
**G7**	Gisum	13
**R26**	Rezvanshahr	16
