# Supplementary material for: Enhanced detection of equine strongyles: Insights from morphological and nemabiome metabarcoding approaches in northern Iran
Source: Equine Vet J. 2025 Nov 29;58(2):508–22. doi: 10.1111/evj.70120 (PMC12892384; doi:10.1111/evj.70120)
Supplement: Supplementary file 2 — Table S2: The number of raw pairs of reads generated by the Illumina MiSeq and the number left at each stage of the metabarcoding bioinformatics pipeline for pools of ~2500 L3s isolated from faeces of horses in Gisum (G), Taleqan (A), Rezvanshahr (R), Tehran resident (T) and Tehran non‐resident (Th). The percentage of retained amplicons assigned to a species with ≥80 bootstrap support, and the number of species identified, are also presented. [file EVJ-58-508-s002.pdf]

Equine Veterinary Journal BEVA

Table S2: The number of raw pairs of reads generated by the Illumina MiSeq and the number left at each stage of the metabarcoding bioinformatics pipeline for pools of ~2500 L3s isolated from faeces of horses in Gisum (G), Taleqan (A), Rezvanshahr (R), Tehran resident (T), and Tehran non-resident (Th). The percentage of retained amplicons assigned to a species with ≥ 80 bootstrap support, and the number of species identified, are also presented.

Sample ID	Raw reads	filtered	denoised forward	denoised reverse	merged	NON-CHIMERIC	Retained Amplicons Assigned ≥ 80%	% Retained Amplicons Assigned ≥ 80%	n Species ≥ 80%
**G1**	24051	23332	23309	23264	23144	22545	22199	98.47	20
**G2**	20585	20032	20026	19997	19950	19433	18279	94.06	24
**A1**	17814	17296	17294	17266	17241	17030	16405	96.33	11
**A2**	40589	39406	39379	39258	39122	37797	36122	95.57	13
**A3**	30790	29995	29971	29916	29778	27998	27035	96.56	12
**A4**	28944	28003	27971	27909	27790	26511	25447	95.99	14
**R1**	27511	26644	26597	26512	26241	23975	23411	97.65	24
**R10**	30986	30046	29991	29921	29601	27201	26414	97.11	28
**R2**	38367	37294	37240	37192	36923	34615	34107	98.53	29
**R3**	29880	28965	28894	28843	28481	26113	25434	97.40	27
**R4**	19860	19164	19128	19051	18726	17879	17660	98.78	24
**R5**	33222	32336	32287	32232	31905	29464	28834	97.86	25

**R6**	21735	21070	21028	21000	20863	19520	19058	97.63	22
**R7**	18139	17614	17598	17555	17471	16446	15478	94.11	17
**R8**	24943	24208	24181	24145	23954	22003	21443	97.45	21
**R9**	30023	29185	29152	29070	28799	28018	27421	97.87	25
**Th10**	22010	21321	21289	21256	21034	20492	20224	98.69	22
**Th11**	26121	25399	25221	25320	24972	24603	24411	99.22	10
**T2**	18720	18135	18124	18062	18038	17575	17542	99.81	13
**Th4**	33436	32523	32491	32388	31943	30421	30070	98.85	28
**Th5**	34302	33237	33204	33095	32768	31551	31097	98.56	25
**Th6**	23823	23070	23025	22962	22715	22310	22032	98.75	23
**Th7**	22622	21892	21872	21825	21727	20088	19706	98.10	17
**T1**	21618	21017	21007	20982	20955	20560	20361	99.03	6
**Th9**	18536	18061	18026	18020	17844	16296	15741	96.59	24
**Average**	26345.08	25569.80	25532.20	25481.64	25279.40	24017.76	23437.24	97.56	20.16
**Std. dev.**	6481.07	6304.45	6296.43	6277.53	6214.39	5837.07	5702.09	1.49	6.48
