# Supplementary material for: Enhanced detection of equine strongyles: Insights from morphological and nemabiome metabarcoding approaches in northern Iran
Source: Equine Vet J. 2025 Nov 29;58(2):508–22. doi: 10.1111/evj.70120 (PMC12892384; doi:10.1111/evj.70120)
Supplement: Supplementary file 3 — Table S3: Prevalence of 32 strongyle species identified using nemabiome metabarcoding for 25 faecal samples from four locations and including both resident and non‐resident horses at a horse‐riding club in Tehran, Iran. [file EVJ-58-508-s004.pdf]

Equine Veterinary Journal BEVA

Table S3: Prevalence of 32 strongyle species identified using nemabiome metabarcoding for 25 fecal samples from four locations and including both resident and non-resident horses at a horse-riding club in Tehran, Iran.

	Location				
	Gisum	Rezvanshahr	Taleqan	Tehran (resident)	Tehran (non-resident)
**Species**	Prevalence (%)	Prevalence (%)	Prevalence (%)	Prevalence (%)	Prevalence (%)
Coronocyclus coronatus	100	100	100	50	71.4
Coronocyclus labiatus	100	100	100	0	85.7
Coronocyclus labratus	100	100	50	0	100
Craterostomum acuticaudatum	0	30	0	0	28.5
Cyathostomum catinatum	100	100	100	50	100
Cyathostomum pateratum	100	100	0	50	85.7
Cylicocyclus ashworthi	100	100	100	50	85.7
Cylicocyclus brevicapsulatus	100	40	0	0	0
Cylicocyclus elongatus	50	60	0	0	14.3
Cylicocyclus insigne	100	100	0	100	100
Cylicocyclus leptostomum	100	100	75	50	100
Cylicocyclus nassatus	100	100	100	100	85.7

Cylicocyclus radiatus	50	90	25	0	57.1
Cylicodontophorus bicoronatus	0	90	0	0	71.4
Cylicostephanus bidentatus	0	10	0	0	14.3
Cylicostephanus calicatus	100	90	100	0	85.7
Cylicostephanus goldi	100	100	100	50	100
Cylicostephanus longibursatus	100	100	100	100	100
Cylicostephanus minutus	100	100	100	100	100
Gyalocephalus capitatus	0	40	0	0	28.5
Parapoteriostomum euproctus	100	70	0	0	57.1
Parapoteriostomum mettami	0	0	0	0	28.6
Petrovinema poculatum	100	50	0	0	42.8
Poteriostomum imparidentatum	0	30	0	0	28.6
Poteriostomum ratzii	0	20	0	0	57.1
Strongylus edentatus	100	80	0	100	100
Strongylus equinus	100	80	0	0	57.1
Strongylus vulgaris	100	100	100	50	100

Triodontophorus brevicauda	50	60	100	0	85.7
Triodontophorus nipponicus	0	60	0	0	28.6
Triodontophorus serratus	50	90	0	50	85.7
Triodontophorus tenuicollis	100	90	0	50	42.8
