# Supplementary material for: Enhanced detection of equine strongyles: Insights from morphological and nemabiome metabarcoding approaches in northern Iran
Source: Equine Vet J. 2025 Nov 29;58(2):508–22. doi: 10.1111/evj.70120 (PMC12892384; doi:10.1111/evj.70120)
Supplement: Supplementary file 4 — Table S4: Pairwise comparisons of species richness and Shannon diversity by locations and methods, estimated using estimated marginal means. The direction of each estimate is relative to the first level in the pair. Bolded values indicate statistically significant comparisons. [file EVJ-58-508-s001.pdf]

Table S4: Pairwise comparisons of species richness and Shannon diversity by locations and methods, estimated using estimated marginal means. The direction of each estimate is relative to the first level in the pair. Bolded values indicate statistically significant comparisons.

	Species richness				Shannon diversity			
**Pairs**	**Estimate**	**Std error**	**z ratio**	***p*-value**	**Estimate**	**Std error**	**t ratio**	***p*-value**
**Gisum - Rezvanshahr**	-0.199	0.148	-1.353	0.657	-0.426	0.154	-2.758	0.065
**Gisum - Taleqan**	0.432	0.204	2.116	0.213	0.522	0.211	2.468	0.121
**Gisum - Tehran:Non-resident**	0.028	0.171	0.164	0.999	-0.114	0.193	-0.592	0.975
**Gisum - Tehran:Resident**	0.744	0.268	2.780	**0.043**	1.632	0.253	6.461	**<0.001**
**Rezvanshahr - Taleqan**	0.632	0.159	3.968	**<0.001**	0.948	0.167	5.686	**<0.001**
**Rezvanshahr - Tehran:Non-resident**	0.228	0.118	1.931	0.300	0.312	0.146	2.136	0.228

**Rezvanshahr - Tehran:Resident**	0.944	0.244	3.862	**<0.001**	2.059	0.225	9.140	**<0.001**
**Taleqan - Tehran:Non-resident**	-0.404	0.172	-2.348	0.130	-0.636	0.181	-3.507	**0.0104**
**Taleqan - Tehran:Resident**	0.312	0.278	1.121	0.796	1.111	0.255	4.354	**<0.001**
**Tehran:Non-resident - Tehran:Resident**	0.7161	0.253	2.828	**0.0377**	1.747	0.237	7.381	**<0.001**
**Morphology - Nemabiome**	-0.819	0.096	-8.504	**<0.001**	-0.655	0.105	-6.217	**<0.001**
